# Long-term oral administration of an HNF4α agonist prevents weight gain and hepatic steatosis by promoting increased mitochondrial mass and function

**DOI:** 10.1038/s41419-022-04521-5

**Published:** 2022-01-27

**Authors:** Vimal Veeriah, Seung-Hee Lee, Fred Levine

**Affiliations:** SBP Medical Discovery Institute, La Jolla, CA 92037 USA

**Keywords:** Cell biology, Cell growth

## Abstract

We report here that the potent HNF4α agonist N-trans-caffeoyltyramine (NCT) promotes weight loss by inducing an increase in mitochondrial mass and function, including fatty acid oxidation. Previously, we found in a short term trial in obese mice that NCT promoted reversal of hepatic steatosis through a mechanism involving the stimulation of lipophagy by dihydroceramides. NCT led to increased dihydroceramide levels by inhibiting dihydroceramide conversion to ceramides. Here, we were able to administer NCT orally, permitting longer term administration. Mice fed NCT mixed with high fat diet exhibited decreased weight. Examination of RNA-seq data revealed an increase in PPARGC1A, a central regulator of mitochondrial biogenesis. In addition to the decreased hepatic steatosis that we found previously, mice fed a high fat diet containing NCT mice weighed substantially less than control mice fed high fat diet alone. They had increased mitochondrial mass, exhibited increased fatty acid oxidation, and had an increased level of NAD. Markers of liver inflammation such as interleukin-6 (IL-6) and tumor necrosis factor alpha (TNFα), which are important in the progression of non-alcoholic fatty liver disease to non-alcoholic steatohepatitis were decreased by NCT. There was no evidence of any toxicity from NCT consumption. These results indicate that HNF4α is an important regulator of mitochondrial mass and function and support that use of HNF4α to treat disorders of fatty acid excess, potentially including obesity, NAFLD, and NASH.

## Introduction

HNF4α is a nuclear receptor transcription factor that controls the expression of downstream genes that are important in multiple aspects of cellular metabolism [1]. The classical view of HNF4α has been that its ligand binding pocket (LBP) is constitutively occupied by a fatty acid that plays a structural rather than regulatory role [2, 3]. However, it has been shown recently that the fatty acids in the HNF4α LBP are exchangeable in the context of full length HNF4α, particularly inside the cell [4]. Using a cell-based promoter reporter assay for human insulin promoter activity that is highly sensitive to HNF4α activity, we demonstrated that fatty acids act as HNF4α antagonists [5]. Using the assay in high-throughput screening, we discovered HNF4α antagonists [5] and more recently agonizts [6]. Most recently, we discovered a potent agonist, N-trans caffeoyltyramine (NCT) [7]. The availability of a potent HNF4α agonist facilitated in vivo studies that have revealed novel aspects of HNF4α biology.

Previously, we showed that intraperitoneal (IP) administration of NCT reversed hepatic steatosis in obese mice fed a high fat diet (HFD) through a previously unsuspected pathway involving regulation by HNF4α of dihydroceramide synthesis. Dihydroceramides were found to control hepatic lipophagy, leading to reversal of hepatic steatosis [7].

Here, we uncovered another previously unsuspected effect of HNF4α that was made possible by the finding that it could be delivered orally mixed with HFD, allowing for longer term NCT administration. As with our previous studies with IP NCT administration [7], there was decreased hepatic steatosis. However, mice on HFD that were administered NCT for 10 weeks had a much lower weight than mice on HFD alone. Decreased fat appeared to be due to an increase in fatty acid oxidation, which in turn was due to increased mitochondrial mass. Consistent with that, there was increased expression of mitochondrial proteins including VDAC1 and electron transport chain proteins, mitochondrial DNA, and total cellular NAD, most of which is in the mitochondria [8]. There was a significant decrease in markers of inflammation and cellular stress, including nitric oxide. Thus, NCT is a strong candidate for a drug that can maintain metabolic homeostasis in the face of challenge from excess fatty acid intake.

## Results

### Long term administration of NCT led to decreased body weight

Previously, we conducted a short, 2-week trial in which NCT, a potent HNF4α agonist, was administered to obese mice. NCT induced decreased hepatic steatosis due to stimulation of lipophagy, but there was no effect on body weight [7]. In that study, NCT was administered intraperitoneally (IP) because HNF4α ligands, including the natural fatty acid ligands and the non-natural ligands that we have studied [5–7] are hydrophobic. That led to compound precipitation when we attempted to deliver NCT subcutaneously (SQ) (Supplementary Fig. 1A–D) or by oral gavage (Supplementary Fig. 1E–H) for 2 weeks. Following oral gavage, NCT was present in stool but not serum (Supplementary Fig. 1I) indicating malabsorption of compound that left the stomach (Supplementary Fig. 1F).

Given the desirability of oral administration, we tested delivery of NCT mixed with HFD mouse chow (Research Diets). HFD containing 4000 ppm NCT (HFD + NCT) (Research Diets), was calculated to provide to a lean mouse weighing approximately 20 g about the same dose of NCT (400 mg/kg/day) as was delivered using the IP administration protocol that we used previously [7].

**Fig. 1:**
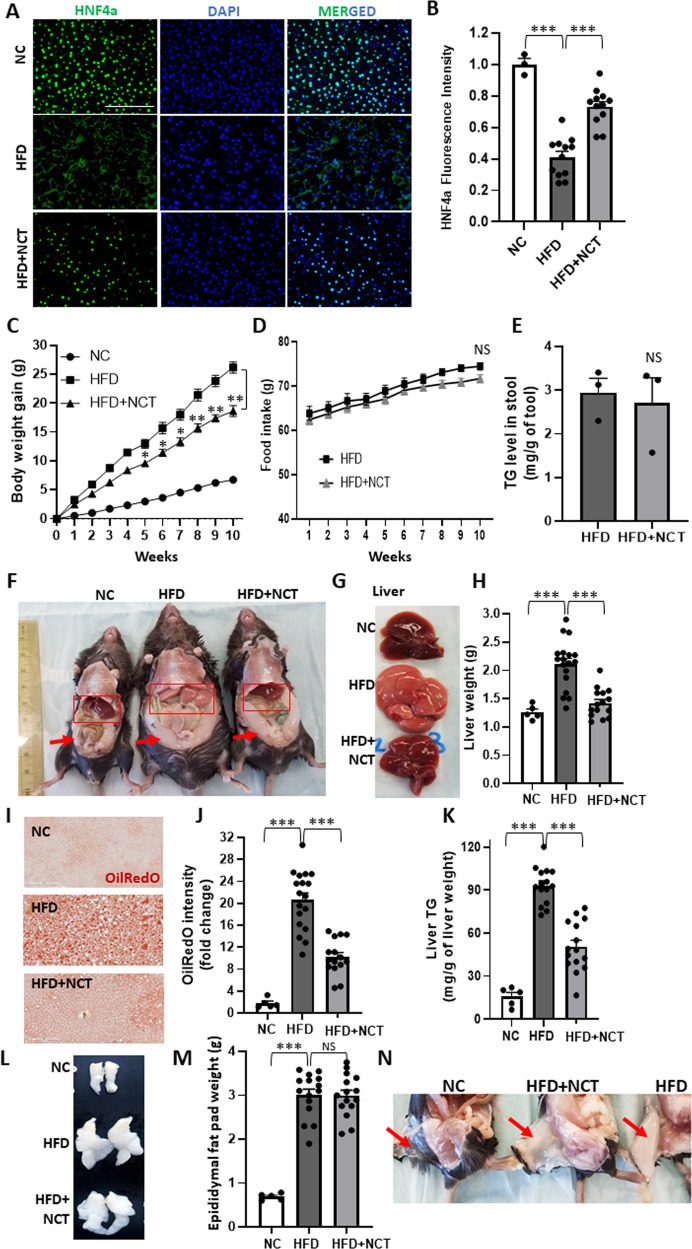
NCT reduced HFD induced weight gain and hepatic steatosis. C57BL/6 mice were fed normal chow (NC), HFD, or HFD + NCT for 10 weeks. **A** Liver sections from mice fed normal chow (NC), high fat diet (HFD) or HFD + NCT were immunostained for HNF4α (green nuclear staining) and DAPI (blue nuclear staining). **B** Quantification of HNF4α fluorescence intensity from images stained as in **A**. Non-specific cytoplasmic staining was subtracted from the HNF4α nuclear staining and fold change was calculated vs. NC (NC, *N* = 3, HFD and HFD + NCT, *N* = 12). **C** Body weight was measured each week for 10 weeks. Body weight gain was calculated by subtracting the baseline body weight at the start of the study (NC *N* = 5, HFD and HFD + NCT, *N* = 15). **D** HFD and HFD + NCT chow consumption per cage was measured every week for 10 weeks, demonstrating no difference between the two groups (five cages for each condition, three mice in each cage). **E** Stool TG was normalized to the stool dry weight (*N* = 3). **F** Photographs of mice from each treatment group, demonstrating reduction of liver size and increased redness with NCT, as well as visceral adiposity. Red box indicates liver and arrow indicates epididymal fat pad. **G** Photographs of dissected livers demonstrating reduction of liver size and increased redness with NCT. **H** Liver weight was normalized to the body weight (NC, *N* = 5, HFD and HFD + NCT, *N* = 15). **I** Photomicrographs of liver sections stained with Oil red O. **J** Quantification of Oil red O staining HFD and HFD + NCT values were measured using image J with consistent threshold settings and normalized to NC values to calculate fold change (NC, *N* = 5, HFD and HFD + NCT, *N* = 15). **K** Hepatic TG level was normalized to the liver weight (NC, *N* = 5, HFD and HFD + NCT, *N* = 15). **L** Representative pictures of epididymal fat pads from each group. **M** Epididymal fat pad weight was quantified by normalizing with total mouse body weight (NC, *N* = 5, HFD and HFD + NCT, *N* = 15). **N** Photographs of mice from each treatment group, demonstrating reduction of subcutaneous fat (arrow indicated). Dots indicate individual mice. Values represent the mean ± SEM. *p < 0.05, **p < 0.01, ***p < 0.001 (HFD vs. NC or HFD + NCT). NS non-significant. Scale bar = 200 μM.

HFD + NCT was administered to 6-week-old C57BL/6 male mice for 10 weeks. To determine whether orally administered NCT was active, we examined for effect on hepatic HNF4α expression, which we found previously to be increased by NCT [7]. HFD led to decreased expression of HNF4α and, consistent with our previous results in which NCT was administered IP [7], this was reversed by NCT (Fig. 1A, B). Thus, oral administration of NCT at approximately the same dose as IP administration was effective at stimulating HNF4α activity.

In striking contrast to our previous experiments with 2 weeks of IP NCT administration, where we did not observe any effect of NCT on body weight, it became evident at week 5 that mice fed HFD + NCT weighed less than mice fed HFD alone (Fig. 1C). By the end of the experiment at 10 weeks, the mice fed HFD + NCT weighed ~10 g less than the mice fed HFD, a 35–40% difference in body weight.

One possible explanation for lower weight was that the mice consumed less chow, possibly due to long term toxicity of NCT. There was no discernible difference in physical activity or other behaviors between mice fed HFD and mice fed HFD + NCT (Supplementary Table 1). Caspase 3, a marker of apoptosis, was low in mice fed HFD, and there was no change with NCT (Supplementary Fig. 2). There was no difference in the amount of HFD chow and HFD + NCT chow that was consumed, indicating that the NCT was not aversive and did not cause the mice to become ill and consume less chow (Fig. 1D). Stool triglyceride (TG) was equal in the HFD and HFD + NCT groups, ruling out the possibility of fat malabsorption as being responsible for the difference in weight (Fig. 1E).

The HFD + NCT mice were markedly less obese (Fig. 1F), with substantially less subcutaneous fat (Fig. 1N) but no change in epididymal fat pad weight at the end of the study (Fig. 1L, M). HFD + NCT mice had redder livers (Fig. 1G), and lower liver weight (Fig. 1H). Consistent with the increased redness and decreased weight, the HFD + NCT livers exhibited decreased Oil Red O staining (Fig. 1I, quantified in J), and lower TG (Fig. 1K). There was no difference in circulating TG or free fatty acid (FFA) (Supplementary Fig. 3).

### NCT led to increased fatty acid oxidation (FAO) in the livers of obese mice with hepatic steatosis but this was a secondary rather than primary effect

The large weight difference between the HFD and HFD + NCT groups in conjunction with decreased subcutaneous and hepatic adiposity was striking. There are only two routes of fat elimination; in the stool by malabsorption, which we had ruled out (Fig. 1E) and by oxidation. Thus, we hypothesized that NCT was inducing an increase in FAO.

Quantification of FAO was done using an assay that measures NADH by the conversion of iodonitrotetrazolium (INT) to INT-formazan mediated by the NADH-requiring enzyme diaphorase [9]. Performing the assay in the presence and absence of added octanoyl-CoA, which is converted through fatty acid β-oxidation to acetyl-CoA with conversion of NAD+ to NADH, provides specificity for FAO [10]. The level of fatty acid oxidation is determined as the INT-formazan level in the presence of octanoyl CoA minus the level in the absence of octanoyl CoA.

**Fig. 2:**
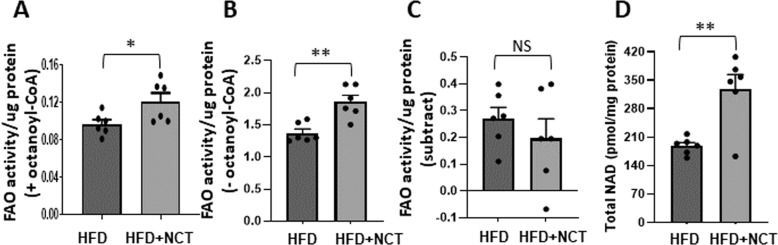
NCT increased hepatic mitochondrial mass. **A** FAO activity in liver lysate prepared in the presence of octanoyl-CoA. **B** FAO activity prepared in the absence of octanoyl-CoA. **C** FAO activity + octanoyl CoA minus FAO activity – octanoyl CoA. **D** Total hepatic NAD level. Dots indicate individual mice (*N* = 6). Values represent the mean ± SEM. *p < 0.05, **p < 0.01 (HFD vs HFD + NCT). NS non-significant.

NCT induced an increase in FAO activity in the presence of octanoyl CoA (Fig. 2A). However, there was also an increase in the baseline activity in the absence of octanoyl CoA (Fig. 2B), so that the overall activity in the assay was unchanged (Fig. 2C). Because this assay is sensitive to the cellular mitochondrial mass, this suggested that the effect of NCT might be on mitochondrial mass rather than specifically on fatty acid oxidation, i.e., NCT might be acting to stimulate increased mitochondrial mass with a consequent increase in total fatty acid oxidation without stimulating an increase in the level of fatty acid oxidation per unit of mitochondrial mass. Independent measurement of total cellular NAD, the majority of which is in the mitochondria [8], revealed a substantial increase in the livers of mice treated with NCT (Fig. 2D). Thus, the effect of NCT on FAO appeared to be a secondary rather than primary effect.

### NCT induced an increase in mitochondrial mass and reduction in mitochondrial stress

**Fig. 3:**
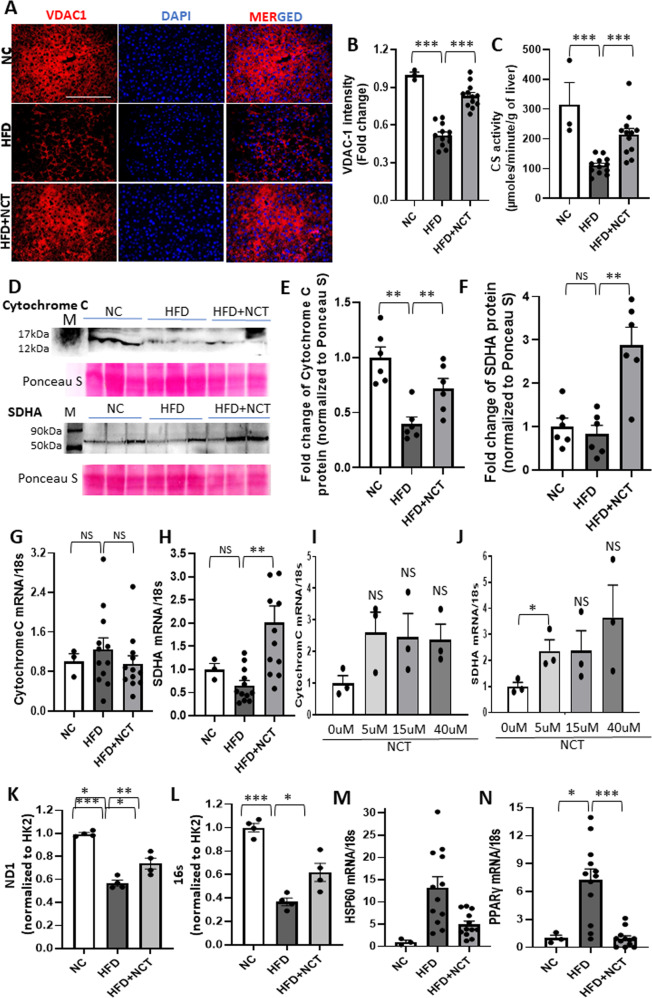
NCT increased mitochondrial mass in mouse liver human primary hepatocyte. **A** Liver sections from NC, HFD, and HFD + NCT mice were immunostained for VDAC-1 (red color). DAPI (blue) is for nuclear staining. **B** Quantification of hepatic VDAC-1 staining intensity (fold change vs. NC, NC, *N* = 3, HFD and HFD + NCT, *N* = 12). **C** Citrate synthase (CS) activity in liver lysate was reduced by HFD, which was reversed by NCT (NC, *N* = 3, HFD and HFD + NCT, *N* = 12). **D** Western blots for cytochrome C and SDHA. After detecting each protein, the membrane was stained for Ponceau S as a control for protein loading. **E**, **F** Quantification of cytochrome C and SDHA protein expression, respectively, in mouse liver. Each value was first normalized to the Ponceau S staining and the fold change was then calculated vs. NC (*N* = 6). **G**, **H** qPCR analysis in mouse liver for *cytochrome C* and *SDHA* mRNA expression normalized with 18s rRNA (NC, *N* = 3, HFD and HFD + NCT, *N* = 12). **I**, **J** qPCR analysis of *cytochrome C* and *SDHA* mRNA from human primary hepatocytes cultured in the indicated concentration of NCT (0, 5, 15, and 40 μM). Values were normalized with 18s rRNA (*N* = 3). **K**, **L** qPCR analysis in mouse liver for mitochondrial DNA (ND1, 16s) expression normalized with HK2 (*N* = 4). **M** qPCR analysis in mouse liver for *HSP60* mRNA expression normalized with 18s rRNA (NC, *N* = 3, HFD and HFD + NCT, *N* = 12). **N** qPCR analysis in mouse liver for *PPARγ* mRNA expression normalized with 18s rRNA (NC, *N* = 3, HFD and HFD + NCT, *N* = 12). Each dot indicates an individual mouse or human donor. Values represent the mean ± SEM. *p < 0.05, **p < 0.01, ***p < 0.001 (HFD vs. NC or HFD + NCT, 0 μM vs. each concentration of NCT in human hepatocyte). NS non-significant. Scale bar = 200 μM.

Based on the FAO oxidation result and increased total cellular NAD, we proceeded to study markers of mitochondrial mass to determine directly whether NCT effected an increase. The two most commonly used protein markers of mitochondrial mass are VDAC1, which is located on the outer mitochondrial membrane [11], and citrate synthase, which is located in the mitochondrial matrix [12]. HFD led to a large decrease in VDAC1 expression and citrate synthase activity, with substantial reversal of those decreases by NCT (Fig. 3A–C).

The major function of mitochondria is oxidative phosphorylation. To determine whether the increased mitochondrial mass was reflected in increased expression of proteins involved in oxidative phosphorylation, we measured cytochrome C and succinate dehydrogenase expression, both of which are important components of the respiratory. Cytochrome C plays a dual role: in mitochondria, being critical for mitochondrial respiration but also playing a role in cell survival [13]. Succinate dehydrogenase, encoded by the SDHA gene, is the catalytic subunit of succinate-ubiquinone oxidoreductase, a complex of the mitochondrial respiratory chain. HFD decreased hepatic cytochrome C and succinate dehydrogenase protein levels and this was reversed by NCT (Fig. 3D–F). SDHA but not cytochrome C mRNA was increased by NCT in mouse liver (Fig. 3G, H). This was reflected in studies with primary human hepatocytes, where NCT increased the level of SDHA but not cytochrome C mRNA (Fig. 3I, J).

An increase in mitochondrial proteins could be due to an increase in the number of mitochondria or simply to an increase in mitochondrial size. Thus, we measured the mitochondrial DNA content, finding that NCT induced a significant increase in mitochondrial DNA, as measured by the level of DNA encoding the mitochondrial genes ND1 and 16S rRNA (Fig. 3K, L).

An important feature of NAFLD and its progression to NASH is mitochondrial stress [14]. To determine whether the reduction in hepatic steatosis and increase in mitochondrial mass induced by NCT translated to reduced mitochondrial stress, we examined the expression of HSP60, a mitochondrial chaperone that is induced by mitochondrial stress, including HFD [15] (Fig. 3M). The level of HSP60 mRNA was greatly decreased by NCT, demonstrating alleviation of mitochondrial stress (Fig. 3M). Similarly, PPARγ plays an important role in the mitochondrial stress response, being activated by fatty acids and exhibiting high expression in fatty liver disease [16]. NCT administration dramatically reduced PPARγ expression to the level in the livers of mice fed normal chow (Fig. 3N).

### NCT increased the activity of the PPARGC1A pathway

**Fig. 4:**
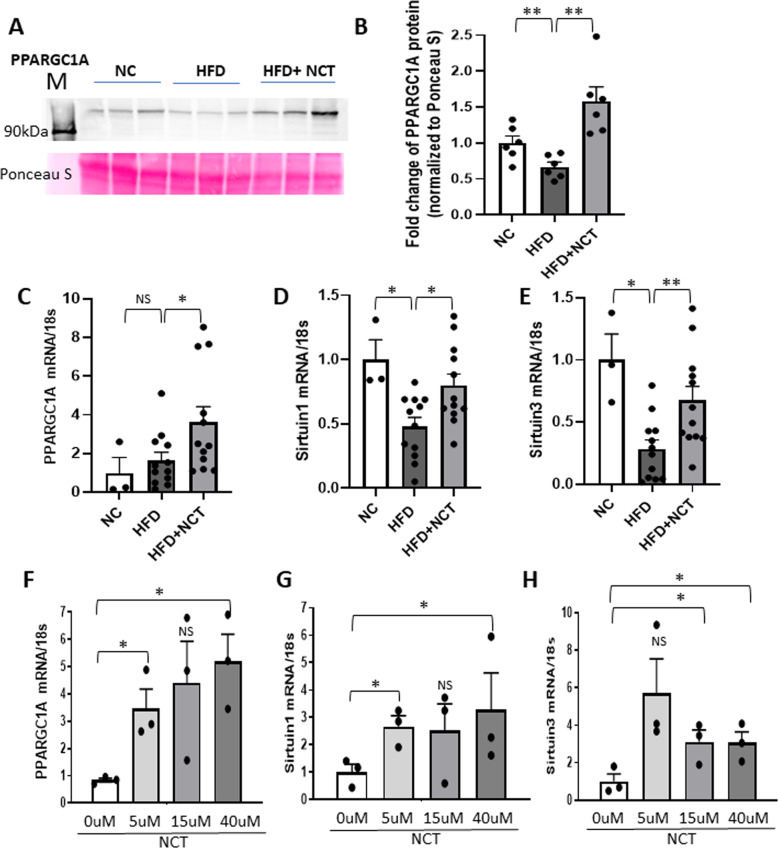
NCT increased PPARGC1A signaling in mouse livers and human primary hepatocytes. **A** Representative pictures of western blot analysis for PPARGC1A expression and Ponceau S in mouse liver. **B** PPARGC1A protein expression (fold change vs. NC) in mouse liver was normalized with Ponceau S (*N* = 6). **C**–**E** qPCR analysis in mouse liver of *PPARGC1A*, *Sirtuin1*, *Sirtuin3* mRNA level normalized with 18s rRNA (NC, *N* = 3, HFD and HFD + NCT, *N* = 12). **F**–**H** qPCR analysis of *PPARGC1A*, *Sirtuin1*, and *Sirtuin3* mRNA level normalized with 18s rRNA in human primary hepatocytes (*N* = 3). Dots indicate individual mouse or human donors. Values represent the mean ± SEM. *p < 0.05, **p < 0.01 (HFD vs. NC or HFD + NCT, 0 μM vs. each concentration of NCT in human hepatocyte). NS non-significant.

The ability of NCT to increase mitochondrial mass raised the question of the mechanism by which that occurred. Examination of RNA-seq data from the livers of mice treated for 2 weeks with NCT revealed an approximately 6-fold increase in *PPARGC1A* (*PGC1a*) mRNA (GEO 174848). PPARGC1A plays an important role in mitochondrial biogenesis but is not known to be regulated by HNF4α. After 10 weeks of oral NCT administration, PPARGC1A protein and mRNA were increased (Fig. 4A–C) in mouse liver. The *PPARGC1A* mRNA level in primary human hepatocytes cultured with NCT was also increased (Fig. 4F).

PPARGC1A activity is controlled by sirtuins, which are NAD-dependent deacetylases. They are both downstream targets of transcriptional activation by PPARGC1A [17] and activators of PPARGC1A activity through deacetylation [18, 19]. HFD reduced *Sirt1* and *Sirt3* mRNA levels, consistent with previous studies [20, 21]. NCT almost completely reversed the effect of HFD on sirtuin gene expression in mouse liver (Fig. 4D, E) and had a significant effect in primary human hepatocytes (Fig. 4G, H). Poly(ADP-ribose) polymerases (PARPs) inhibit mitochondrial function and PPARGC1A activity [22]. In contrast to sirtuin expression, *Parp1* and *Parp2* mRNA levels were not modulated by NCT (Supplementary Fig. 4).

### NCT inhibited inflammation

**Fig. 5:**
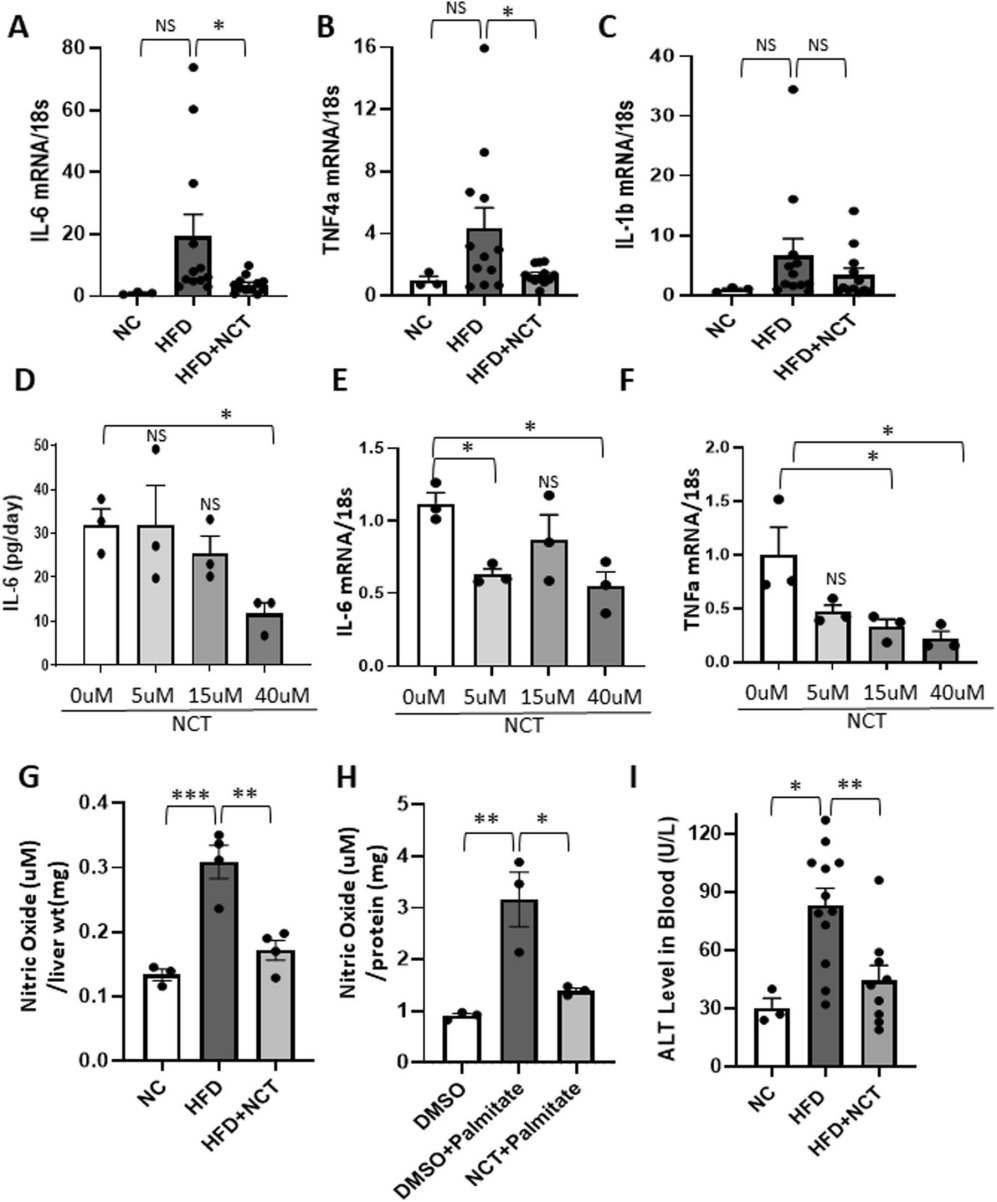
NCT reduced inflammatory markers in mouse livers and human primary hepatocytes. **A**–**C** qPCR analysis in mouse liver of *IL-6*, *TNF*α, and *IL-1β* mRNA levels normalized to 18s rRNA (NC, *N* = 3, HFD and HFD + NCT, *N* = 12). **D** ELISA analysis for IL-6 secreted into the medium of human primary hepatocytes cultured in palmitate and DMSO (vehicle control) or different concentrations of NCT (*N* = 3). **E**, **F** qPCR analysis of *IL-6* and *TNF*α mRNA level in primary human hepatocytes. Normalization was to 18s rRNA (*N* = 3). **G**, **H** Nitric oxide (NO) analysis in in vivo and in vitro. NO expression normalized to liver weight in vivo (NC, *N* = 3; HFD and HFD + NCT, *N* = 4) and normalized to protein in T6PNE cells in vitro (*N* = 3). **I** Alanine aminotransferase (ALT) level in blood was reduced in HFD + NCT mice compared to HFD mice (NC, *N* = 3; HFD, *N* = 12; HFD + NCT, *N* = 9). Dots indicate individual mouse or human donors. Values represent the mean ± SEM. *p < 0.05, **p < 0.01, ***p < 0.001 (HFD vs. NC or HFD + NCT, 0 μM vs. each concentration of NCT in human hepatocyte and T6PNE cells). NS non-significant.

Inflammation is a major factor in the pathophysiology of NAFLD and its progression to NASH [14]. Inflammation contributes to essential features of NASH pathology, including fibrosis and hepatocyte death, ultimately leading to cirrhosis. *IL-6* and *TNF*α are inflammatory mediators that are important in NASH [14]. Both were significantly reduced by NCT in the livers of HFD + NCT mice compared to HFD mouse livers (Fig. 5A, B). Similarly, *IL-6* mRNA and protein were reduced by NCT in cultured human hepatocytes (Fig. 5D, E). *TNF*α mRNA was also decreased in a dose-responsive manner by NCT in cultured human hepatocytes (Fig. 5F) but *IL1β* expression was not significantly changed (Fig. 5C). Nitric oxide (NO) plays an important role in inflammatory responses [23], including NAFLD [24]. NO was increased in the livers of mice fed HFD (Fig. 5G) as well as in cultured cells treated with palmitate (Fig. 5H) and was decreased by NCT in both settings (Fig. 5G, H). The blood ALT level was elevated in mice fed HFD and was significantly decreased by NCT (Fig. 5I). The blood ALP was unchanged (Supplementary Fig. 3C). Other components of the liver profile panel and hematological analysis showed no difference in HFD versus HFD + NCT mice (Supplementary Tables 2 and 3).

## Discussion

The principal finding of the studies presented here is that activating HNF4α with the potent HNF4α agonist NCT led to decreased weight in mice fed a high-fat diet. This was due to a substantial increase in mitochondrial mass and consequent increase in fatty acid oxidation. Our earlier studies where NCT was administered to mice for a shorter time found a different effect, i.e., stimulation of lipophagy. That led to decreased hepatic steatosis but no change in weight, as the fatty acids released from lipid vesicle by lipophagy appeared to redistribute to adipose tissue. Thus, there appear to be at least two independent effects of HNF4α agonism on fat storage: an immediate effect on through lipophagy and a longer-term effect on mitochondrial mass leading to increased mitochondrial function, including fatty acid oxidation.

A key to uncovering the effect of pharmacological activation of HNF4α on mitochondrial mass was the finding that oral delivery of NCT in high fat chow was effective, permitting long term delivery of the compound. Being able to deliver drugs orally is often critical to achieving effective clinical translation, so this was an important goal. Oral delivery is particularly important for highly prevalent and chronic diseases like NAFLD/NASH. Our previous study of NCT used IP delivery because the compound is hydrophobic, limiting some routes of delivery. However, by combining NCT with high fat chow, we were able to demonstrate efficacy in a HFD diet mouse model of NAFLD. Of note, there is a large difference between rodent and human microsomal stability of NCT, with NCT being much more stable in the presence of human microsomes than with rodent [7]. While this bodes well for eventual use in humans, PK studies in the context of human clinical trials will be required. A potentially important consideration is that it is likely that NCT and fatty acids are in competition for the HNF4α ligand binding pocket. The concentration of fatty acids, which act as HNF4α antagonists [5], in the nucleus, is unknown, but if it is related to the overall level of steatosis in a particular organ, that could affect the dose required for efficacy.

We were led to the increase in mitochondrial mass by the decreased weight in the mice fed HFD + NCT and those fed HFD alone. Since food consumption was the same and there was no fat malabsorption, only an increase in fatty acid oxidation could reasonably account for the lower weight of the HFD + NCT mice. This was borne out by increased oxidation of octanoyl CoA in mice fed HFD + NCT. However, the increase in fatty acid oxidation was not selective, as there was increased NADH production even in the absence of octanoyl CoA. Consistent with a general increase in mitochondrial mass and function, VDAC1 and citrate synthase were increased, as were the oxidative phosphorylation components cytochrome C and SDHA. Consistent with a general increase in mitochondrial function, total cellular NAD was substantially increased by NCT.

The pathways controlling mitochondrial mass are complex and interlocking and a protein at the center of those pathways is the transcriptional coactivator PPARGC1A (PGC1α) [25]. PPARGC1A interacts with multiple factors involved in mitochondrial biogenesis and function. Notably, it interacts directly with HNF4α in regulating gene expression, particularly gluconeogenesis [26], but HNF4α has not heretofore been recognized as having an effect on mitochondrial biogenesis. PPARGC1A expression was increased by NCT but is not thought to be a direct HNF4α target based on ChIP-seq data [27]. Moreover, NCT increased the expression of sirtuins, which control the level of PPARGC1A activity through deacetylation [19]. Pharmacological enhancement of mitochondrial mass and function has been a long-sought goal, but there have been no robustly active compounds with the desired activity [28, 29].

A major motivation for the discovery of approaches to increase mitochondrial mass and function is that mitochondrial function is declines with aging [30]. Given our finding of increased mitochondrial mass and function by HNFα activation NCT has the potential to be of benefit in that setting. However, a consideration with stimulating HNF4α activity is that it is expressed at a high level only in a subset of tissues, including the liver, intestine, pancreas, and kidney. Thus, the effects of HNF4α agonizts may be limited to those organs, although there is evidence for low level HNF4α expression elsewhere [31]. Furthermore, despite the fact that HNF4α is not expressed in adipocytes, the effect of NCT extended to decreased adiposity. Furthermore, there are important age-related diseases that affect organs expressing HNF4α. Most prominent among those is type 2 diabetes, in which age and lipotoxic effects on pancreatic β-cells over time are critical [32]. Thus, NCT has potential as a type 2 diabetes therapeutic.

The liver plays a key role in type 2 diabetes, where the predominant paradigm is that cellular stress and inflammation contribute to insulin resistance and dysregulated hepatic gluconeogenesis [33], as well as being important in the progression from NAFLD to NASH. NCT was effective at reducing cellular stress and inflammation, supporting a role in multiple disease in which those factors are central.

Of significance for eventual clinical translation of NCT, we have not seen any deleterious effects of this compound in any of our studies, including a short-term maximum tolerated dose study [7], two-week IP administration at a fairly high dose of 200 mg/kg bid [7], and long term oral administration. Given the chronic nature of disorders of lipotoxicity, the lack of discernable toxicity of NCT is critical, but this will obviously require additional investigation in human clinical trials.

## Materials and methods

### In vivo mouse experiments

#### NCT oral administration

Four week-old male C57BL/6J (JAX cat#000664) mice were purchased from Jackson laboratory and were maintained in a 12 h light/day cycle throughout the experiment. Prior to the experiments, mice were acclimated for 2 weeks. Six-week-old mice with similar body weights were randomized to normal diet (NC), HFD (Research Diets, cat# D12492 60 kcal% fat) or HFD containing 4000 ppm NCT (HFD + NCT) (Research Diets, 60 kcal% fat + 4000 ppm NCT), which was calculated to provide approximately 400 mg/kg/d NCT. HFD chow containing NCT was made with gray dye to distinguish it from regular HFD chow that had green dye. Mice for each treatment group were placed in separate cages. Equal amounts of fresh chow were provided every week to all mice. Body weight gain and food intake were measured every week for 10 weeks. After 10 weeks of chow treatment, mice were sacrificed for analysis. All animal experiments were approved by the Institutional Animal Care and Use Committee (IACUC) of the Sanford Burnham Prebys Medical Discovery Institute in accordance with national regulations. Sample size was chosen based on the prior study of NCT delivered by IP injection [7].

#### Subcutaneous and oral gavage treatment

Twelve-week-old C57BL/6J DIO male mice were purchased from Jackson laboratory (cat#380050) and were fed with HFD (Research Diets, cat# D12492 60 kcal% fat). Prior to the experiments, mice were acclimated for 2 weeks. Fourteen-week-old mice were injected subcutaneously using sterile insulin syringes filled with DMSO or NCT (200 mg/kg of mouse body weight) bid for 2 weeks. For oral gavage, 14-week-old C57BL/6J DIO male mice were fed by oral gavage using a feeding needle attached to a sterile 1 mL syringe filled with 200 μL of methyl cellulose (MC, vehicle control) or 200 μL of NCT (200 mg/kg) dissolved in methyl cellulose twice a day for 2 weeks. All mice were maintained in a 12 h light/day cycle throughout the experiment. For analysis all mice were sacrificed after 2 weeks of treatment.

#### Sample collection

Mouse samples were collected as described previously [34]. Briefly, on the final day of treatment mice received dextrose (3 g/kg of body weight) by IP injection to stimulate insulin secretion, which inhibits FFA release from adipocytes, leaving liver-derived FFA as the major source of circulating FFA. One hour later, blood samples were collected via retro-orbital bleeding and mice were euthanized using pentobarbital. Mice were dissected aseptically and liver, epididymal fat, and body weights were measured and pictures taken. Dissected liver samples were washed immediately in sterile cold PBS and cut into small pieces. Half of the liver samples were snap frozen using liquid nitrogen and stored at −80 °C for RNA, protein isolation, and liver lysate preparations. The other half were fixed in 4% of cold paraformaldehyde (PFA, Santa Cruz Biotechnology, USA) and processed for histomorphometry and immunofluorescence.

#### Oil Red O staining (in vivo) and analysis

Oil red O staining was performed as described previously [34]. Slides containing frozen liver tissue sections from mice were air dried for 10–20 min followed by rehydration in distilled water. Sections were immersed in absolute propylene glycol (Cat# 151957, MP Biomedicals, LLC, USA) for 2 min followed by 0.5% in Oil red O solution (Cat# K043, Poly Scientific R&D, USA) for 2 h. Slides were then differentiated in 85% propylene glycol solution, washed with dH_2_O for 2 h, and mounted using glycerin jelly mounting medium. All slides were scanned at a magnification of ×20 using the Aperio Scanscope FL system (Aperio Technologies Inc., Vista, CA, USA). The liver area stained with oil red O was measured using image J software as described [35], with some modifications as follow, Oil red O-stained liver images were opened in Image J software. Using the *Analyze* > *Set Scale* command, the scale bar of the images was set to 200 µm. RGB images were then converted into gray scale images using the *Image* > *Type* > *RGB Stack* command and were split into red, blue and green channels. Using the *Image* > *Adjust* > *Threshold* command, the threshold was manually set to highlight the Oil red O-stained lipid droplets in the green channel. We used the same threshold for all the images in all treatment groups and the % oil red O-stained area was obtained using the *Analyze* → *Measure* tool command. Fold change was calculated by normalizing the values to images from mice fed normal chow.

#### Triglyceride analysis

The TG level in mouse liver, serum, and stool was measured according to manufacturer’s instructions using the Triglyceride Calorimetric Assay Kit (Cat# 10010303, Cayman Chemicals, USA). Liver and stool TG was normalized with liver and stool weight, respectively. Fold change was calculated by normalizing to values from mice fed NC.

#### Free fatty acid quantification

Blood samples were collected from dextrose injected mice and centrifuged to collect serum samples. The serum FFA level was measured according to manufacturer’s instructions using a Free Fatty Acid Quantification Colorimetric/ Fluorometric Kit (Cat# K612, BioVision, USA). Fold change was calculated by normalizing to values from mice fed NC.

#### Immunofluorescence

Frozen liver sections were permeabilized using 0.3% Triton-X and incubated in antigen retrieval solution (Antigen retrieval citrate, Biogenex) at sub-boiling temperature for 10 min. Subsequently, sections were incubated with blocking buffer containing 5% normal donkey serum (Jackson Immuno Research) followed by incubation overnight at 4 °C with mouse anti-HNF4α monoclonal antibody (1:800, Cat# PP-H1415–00, R&D Systems), rabbit polyclonal anti-VDAC antibody (1:400, cat# PA1–954A, Invitrogen) and cleaved caspase3 antibody (1:500, cat#9664, Cell Signaling). Sections were washed and incubated for 1 h at room temperature with anti-mouse secondary antibody coupled with Alexa fluor 488 (1:400, Invitrogen) and anti-rabbit secondary antibody coupled with rhodamine red. Nuclei were visualized by counterstaining with DAPI (40,6-diamidino-2-phenylindole, Sigma Aldrich). Slides were mounted using fluorescence mounting medium and images were obtained at ×40 magnification using an Olympus IX71 fluorescence microscope. Fluorescence intensity of HNF4α-stained nuclei and VDAC stained mitochondria was calculated using MetaMorph TL software (version 7.6.5.0, Olympus). Fold change was calculated by normalizing to values from mice fed NC.

#### Liver profile analysis

Mice were anesthetized and 100 μL of whole blood was collected via retro-orbital bleeding in lithium heparin blood collection tubes and transferred to single use VetScan mammalian liver profile reagent rotors. The levels of multiple analytes, including alkaline phosphatase (ALP), alanine aminotransferase (ALT), gamma glutamyl transferase (GGT), bile acids (BA), total bilirubin (TBIL), albumin (ALB), blood urea nitrogen (BUN), total cholesterol (CHOL) were quantified using a VetScan VS2 Chemistry Analyzer (Abaxis North America, USA).

#### Blood count analysis

Mice were anesthetized and 20 μL of whole blood was collected via retro-orbital bleeding in lithium heparin blood collection tubes and 20 different hematologic parameters were measured using a Hemavet 950 FS blood count analyzer (Drew Scientific Group).

#### Western blotting

Mouse liver extracts were prepared by incubation in RIPA buffer (Invitrogen) containing protease inhibitors (Calbiochem, San Diego, CA). Protein was quantified by BCA assay (Thermo Scientific). Protein (40 or 80 mg for cytochrome C) was separated on 12 or 16% Tri-Glycine gels (Invitrogen) and transferred to Immobilon P membrane (0.2 μm pore size, Millipore). After 1 h in phosphate-buffered saline Tween (PBST) with 3% milk, membranes were incubated with antibodies to PPARGC1A(PGC1α) (Cat# NBP1-04676, Novus, 1:1000), SDHA XP (Cat#11998, Cell Signaling 1:1000), or Cytochrome C (Cat# 4280, Cell Signaling 1:500), followed by secondary antibody conjugated to horseradish peroxidase (1:5000, Jackson Immune or Cell Signaling). Signal was revealed by ECL (Thermo) and imaged with a ChemiDoc MP imager (Bio-Rad). After detection, membrane was incubated with Ponceau S solution (Sigma) for 1 h for normalization to loaded protein.

#### Primary human hepatocytes

Experiments with primary human hepatocytes were performed by CN-Bio (Cambridge, UK) [36]. Briefly, primary human hepatocytes (PHHs), human Kupffer cells (HKs) and human stellate cells (HSCs) were seeded onto CN-Bio’s PhysioMimix LC12 MPS culture plates at 6 × 10^5^ cells for PHHs and 6 × 10^4^ cells for HKs and HSCs in 1.6 ml of CN-Bio’s HEP-lean media with 5% FCS. Throughout the experiment the cells were maintained at a flow rate of 1 μl/s. After 24 hours (Day 1) of seeding, the media was changed to HEP-lean media and the cells were incubated until day 4 to allow the cells to form microtissues. At day 4 post seeding, media was changed to HEP-fat media and treated with DMSO or NCT (5, 15, and 40 μM). Media was replaced on days 6 and 8. Cells were harvested on day 10 for RNA extraction and culture media was collected for ELISA analysis.

#### RT-PCR

Total RNA was isolated from liver tissues and primary human hepatocytes using Trizol (Invitrogen). cDNA was amplified using 3 μg of total RNA using qScript cDNA SuperMix (Quanta BioSciences, Beverly, MA, USA). Quantitative real time PCR (RT-PCR) analysis was performed using SYBR® Select Master Mix (Applied Biosystems) and an ABI 7900HT thermal cycler (Applied Biosystems, Thermo Fisher Scientific). Ct values were normalized to 18s rRNA and are expressed as fold change over samples from mice fed NC or cultured human hepatocytes without NCT.

#### Fatty acid oxidation assay

FAO in liver lysate was measured according to manufacturer’s instructions using a calorimetric assay kit (Cat# E-141, Biomedical Research Service Center, State University of New York, Buffalo, NY).

#### Nicotinamide adenine dinucleotide assay

Total NAD level in liver lysate was analyzed according to manufacturer’s instructions using a calorimetric NAD+/NADH assay kit (Cat# MET-5014, Cell Biolabs, Inc. USA).

#### Citrate synthase activity

Citrate synthase activity in liver homogenate was measured according to manufacturer’s instructions using a calorimetry based MitoCheck Citrate Synthase Activity Assay Kit (Cat# 701040, Cayman Chemicals, USA).

#### Bicinchoninic acid (BCA) assay

A BCA protein assay was performed according to manufacturer’s instructions using a kit from Thermo Scientific (Cat# 23225). BCA assay was used for protein quantification for the FAO assay and Western blotting. Absorbance at 550 nm was determined using a plate reader.

### Nitric oxide assay

T6PNE cells [6] were maintained in RPMI (5.5 mM glucose, Corning) supplemented with 10% fetal bovine serum (FBS, Sigma-Aldrich) and 1% penicillin–streptomycin (pen-strep, Gibco) in 5% CO2 at 37 °C. Cells were treated with 0.12 μM palmitate plus 0 or 15 μM NCT for 3 days in 10 cm plates and harvested with 500 μL PBS. For tissue specimens, snap frozen mouse liver was weighed and homogenized with PBS. NO was measured with the QuantiChrom Nitric Oxide Assay kit (D2NO-100, BioAssay Systems). Homogenized samples (150 μL) were processed for deproteination with 8 μL ZnSO_4_ and 8 μL NaOH. For normalization, an aliquot of samples from T6PNE cells was taken for BCA assay before deproteination. Samples and standard from kit incubated with reagents for 20 min at 60 °C and measured OD 540 nM.

### Palmitate–BSA complex

Palmitate (150 mM) (Sigma-Aldrich) was prepared in 50% ethanol and precomplexed with 15% fatty acid-free BSA (Research Organics, Cleveland, OH, USA) in a 37 °C water shaker. BSA-precomplexed palmitate was used as a 12 mM stock solution for all assays with a final concentration of 0.12 mM palmitate in cell culture medium.

### Mitochondrial DNA analysis

Quantification of mtDNA was performed as described [37]. Snap frozen mouse liver was homogenized and total cellular DNA was extracted with a QIAamp DNA kit (Qiagen) followed by qPCR with the primers below:

16S rRNA primers FWD: 5′-CCGCAAGGGAAAGATGAAAGAC-3′

REV: 5′-TCGTTTGGTTTCGGGGTTTC-3′

ND1 primers FWD: 5′-CTAGCAGAAACAAACCGGGC-3′

REV: 5′-CCGGCTGCGTATTCTACGTT-3′

HK2 primers FWD: 5′-GCCAGCCTCTCCTGATTTTAGTGT-3′

REV: 5′-GGGAACACAAAAGACCTCTTCTGG-3′

### Statistical analysis

Data are presented as mean ± SEM of three or more samples as indicated. Statistical significance was assessed using Student’s *t*-test or ANOVA.

## Supplementary information

Veeriah, Lee, Levine Supplementary Materials

Reproducibility Checklist

## Data Availability

Unique reagents generated in this study will be made available upon request. An agreement with our institute’s Materials Transfer Agreement (MTA) may be required. Further information and requests for resources and reagents should be directed to Fred Levine (flevine@sbpdiscovery.org).

## References

[1] Yeh MM, Bosch DE, Daoud SS (2019). Role of hepatocyte nuclear factor 4-alpha in gastrointestinal and liver diseases. World J Gastroenterol.

[2] Sladek F (2002). Desperately seeking…something. Mol Cell.

[3] Dhe-Paganon S, Duda K, Iwamoto M, Chi YI, Shoelson SE (2002). Crystal structure of the HNF4 alpha ligand binding domain in complex with endogenous fatty acid ligand. J Biol Chem.

[4] Yuan X, Ta TC, Lin M, Evans JR, Dong Y, Bolotin E (2009). Identification of an endogenous ligand bound to a native orphan nuclear receptor. PLoS ONE.

[5] Kiselyuk A, Lee SH, Farber-Katz S, Zhang M, Athavankar S, Cohen T (2012). HNF4alpha antagonists discovered by a high-throughput screen for modulators of the human insulin promoter. Chem Biol.

[6] Lee SH, Athavankar S, Cohen T, Piran R, Kiselyuk A, Levine F (2013). Identification of alverine and benfluorex as HNF4alpha activators. ACS Chem Biol.

[7] Lee SH, Veeriah V, Levine F (2021). Liver fat storage is controlled by HNF4à through induction of lipophagy and is reversed by a potent HNF4à agonist. Cell Death Dis.

[8] Stein LR, Imai S (2012). The dynamic regulation of NAD metabolism in mitochondria. Trends Endocrinol Metab.

[9] Bergel A, Souppe J, Comtat M (1989). Enzymatic amplification for spectrophotometric and electrochemical assays of NAD+ and NADH. Anal Biochem.

[10] Kwong SC, Jamil AHA, Rhodes A, Taib NA, Chung I (2019). Metabolic role of fatty acid binding protein 7 in mediating triple-negative breast cancer cell death via PPAR-alpha signaling. J lipid Res.

[11] Camara AKS, Zhou Y, Wen PC, Tajkhorshid E, Kwok WM (2017). Mitochondrial VDAC1: a key gatekeeper as potential therapeutic target. Front Physiol.

[12] Larsen S, Nielsen J, Hansen CN, Nielsen LB, Wibrand F, Stride N (2012). Biomarkers of mitochondrial content in skeletal muscle of healthy young human subjects. J Physiol.

[13] Alvarez-Paggi D, Hannibal L, Castro MA, Oviedo-Rouco S, Demicheli V, Tortora V (2017). Multifunctional cytochrome c: learning new tricks from an old dog. Chem Rev.

[14] Schuster S, Cabrera D, Arrese M, Feldstein AE (2018). Triggering and resolution of inflammation in NASH. Nat Rev Gastroenterol Hepatol.

[15] Xiao T, Liang X, Liu H, Zhang F, Meng W, Hu F (2020). Mitochondrial stress protein HSP60 regulates ER stress-induced hepatic lipogenesis. J Mol Endocrinol.

[16] Pettinelli P, Videla LA (2011). Up-regulation of PPAR-gamma mRNA expression in the liver of obese patients: an additional reinforcing lipogenic mechanism to SREBP-1c induction. J Clin Endocrinol Metab.

[17] Kong X, Wang R, Xue Y, Liu X, Zhang H, Chen Y (2010). Sirtuin 3, a new target of PGC-1alpha, plays an important role in the suppression of ROS and mitochondrial biogenesis. PLoS ONE.

[18] Watroba M, Szukiewicz D (2016). The role of sirtuins in aging and age-related diseases. Adv Med Sci.

[19] Nemoto S, Fergusson MM, Finkel T (2005). SIRT1 functionally interacts with the metabolic regulator and transcriptional coactivator PGC-1{alpha}. J Biol Chem.

[20] Deng XQ, Chen LL, Li NX (2007). The expression of SIRT1 in nonalcoholic fatty liver disease induced by high-fat diet in rats. Liver Int.

[21] Cho EH (2014). SIRT3 as a regulator of non-alcoholic fatty liver disease. J Lifestyle Med.

[22] Lu P, Hogan-Cann AD, Kamboj A, Roy Chowdhury SK, Aghanoori MR, Fernyhough P (2019). Poly(ADP-ribose) polymerase-1 inhibits mitochondrial respiration by suppressing PGC-1alpha activity in neurons. Neuropharmacology.

[23] Sharma JN, Al-Omran A, Parvathy SS (2007). Role of nitric oxide in inflammatory diseases. Inflammopharmacology.

[24] Cunningham RP, Sheldon RD, Rector RS (2020). The emerging role of hepatocellular eNOS in Non-alcoholic fatty liver disease development. Front Physiol.

[25] Scarpulla RC (2011). Metabolic control of mitochondrial biogenesis through the PGC-1 family regulatory network. Biochim Biophys Acta.

[26] Rhee J, Inoue Y, Yoon JC, Puigserver P, Fan M, Gonzalez FJ (2003). Regulation of hepatic fasting response by PPARgamma coactivator-1alpha (PGC-1): requirement for hepatocyte nuclear factor 4alpha in gluconeogenesis. Proc Natl Acad Sci USA.

[27] Odom DT, Zizlsperger N, Gordon DB, Bell GW, Rinaldi NJ, Murray HL (2004). Control of pancreas and liver gene expression by HNF transcription factors. Science.

[28] Chavez JD, Tang X, Campbell MD, Reyes G, Kramer PA, Stuppard R (2020). Mitochondrial protein interaction landscape of SS-31. Proc Natl Acad Sci USA.

[29] Whitaker RM, Corum D, Beeson CC, Schnellmann RG (2016). Mitochondrial biogenesis as a pharmacological target: a new approach to acute and chronic diseases. Annu Rev Pharm Toxicol.

[30] Kauppila TES, Kauppila JHK, Larsson NG (2017). Mammalian mitochondria and aging: an update. Cell Metab.

[31] Niehof M, Borlak J (2009). Expression of HNF4alpha in the human and rat choroid plexus: implications for drug transport across the blood-cerebrospinal-fluid (CSF) barrier. BMC Mol Biol.

[32] Janikiewicz J, Hanzelka K, Kozinski K, Kolczynska K, Dobrzyn A (2015). Islet beta-cell failure in type 2 diabetes-Within the network of toxic lipids. Biochem Biophys Res Commun.

[33] Loria P, Lonardo A, Anania F (2013). Liver and diabetes. A vicious circle. Hepatol Res.

[34] Lee SH, Veeriah V, Levine F (2021). Liver fat storage is controlled by HNF4alpha through induction of lipophagy and is reversed by a potent HNF4alpha agonist. Cell Death Dis.

[35] Mehlem A, Hagberg CE, Muhl L, Eriksson U, Falkevall A (2013). Imaging of neutral lipids by oil red O for analyzing the metabolic status in health and disease. Nat Protoc.

[36] Kostrzewski T, Cornforth T, Snow SA, Ouro-Gnao L, Rowe C, Large EM (2017). Three-dimensional perfused human in vitro model of non-alcoholic fatty liver disease. World J Gastroenterol.

[37] Quiros PM, Goyal A, Jha P, Auwerx J (2017). Analysis of mtDNA/nDNA ratio in mice. BCurr Protoc Mouse Biol.

