# Supplementary material for: Long-term oral administration of an HNF4α agonist prevents weight gain and hepatic steatosis by promoting increased mitochondrial mass and function
Source: Cell Death Dis. 2022 Jan 27;13(1):89. doi: 10.1038/s41419-022-04521-5 (PMC8795379; doi:10.1038/s41419-022-04521-5)
Supplement: Supplementary file 1 — Veeriah, Lee, Levine Supplementary Materials [file 41419_2022_4521_MOESM1_ESM.docx]

Veeriah, Lee, and Levine. **Long-term oral administration of an HNF4α agonist prevents weight gain and hepatic steatosis by promoting increased mitochondrial mass and function.**

**SUPPLEMENTARY MATERIALS:**


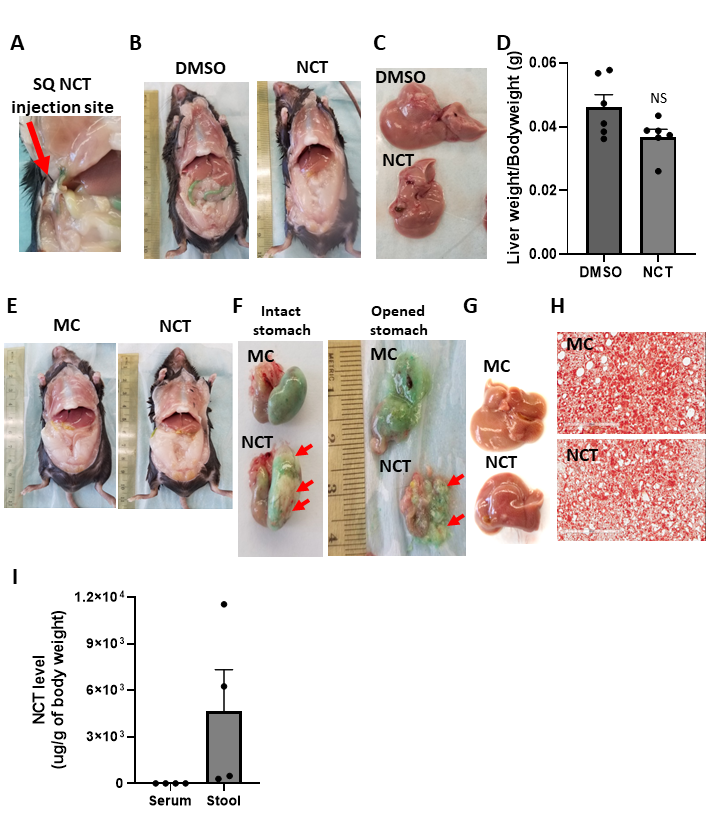


**Supplementary Figure 1: NCT subcutaneous and oral gavage delivery led compound precipitation: A-D.** 14 weeks old male C57BL/6 DIO mice were received 200mg/kg of NCT 2 doses per day for 2 weeks via subcutaneous injection. **A.**  Precipitation of NCT on subcutaneous injection site on representative mouse. Red arrow indicates the compound precipitation. **B.** Representative picture of dissected subcutaneous NCT and DMSO injected mouse. **C.** Dissected liver pictures of representative mice (N=6). No difference in liver color. **D.** Liver weight (normalized with body weight) was measured (N=6). **E-I**.14 weeks old male C57BL/6 DIO mice were received methyl cellulose (MC, vehicle control) or NCT (200mg/kg) 2 doses per day via oral gavage. **E.** Representative picture of dissected methyl cellulose and NCT treated mice (N=5). **F.** Representative picture of dissected stomach of methyl cellulose (MC) and NCT treated mice (N=5). Right: intact stomach Left: opened stomach and red arrows indicates the NCT precipitation inside stomach. **G.** Representative picture of dissected liver. No difference in liver color. **H.** Representative pictures of Oil Red O staining on liver sections. **I.** NCT compound level measured in serum and stool (N=4). Dots indicate individual mice, NS=non-significant. Values represent the mean ± SEM. Scale bar=200μM

**Supplementary Figure 2.** T6PNE cells were treated for 3 days with or without palmitate (0.1mM) and 0, 5, 10, 20μM NCT, followed by fixing with 4% PFA and immunostaining for cleaved caspase3 and DAPI. Quantification was with a Celigo imaging cytometer (Nexcelom Bioscience). **A**. Immunostaining of cleaved caspase3 (red) and DAPI (blue). **B**. Total DAPI number per well. **C**. Quantification of cleaved caspase3 positive cells normalized to cell number measured with DAPI. **D**. Immunostaining of cleaved caspase3 (red), HNF4α (green) and DAPI (blue) in mouse liver. Values represent the mean ± SEM. NS=non-significant. Scale bar=200μM


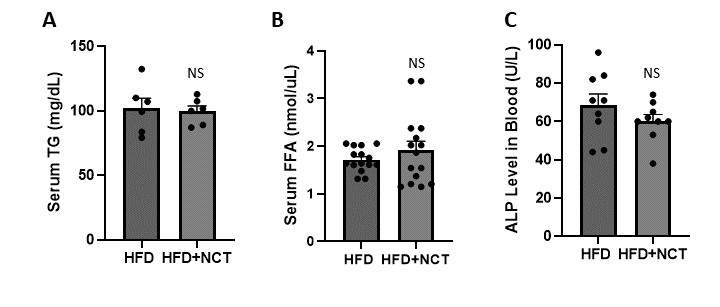


**Supplementary Figure 3: NCT did not modulate the TG, FFA and ALP level in the circulation: A.** TG level in serum samples collected from mice after 10 weeks of HFD and NCT treated HFD chow fed mice was quantified (N=6). **B.** Free fatty acid in serum sample of these mice was quantified (N=15). **C.**  ALP level in the blood samples of these mice was quantified (N=9). Dots indicate individual mice, NS=non-significant. Values represent the mean ± SEM.


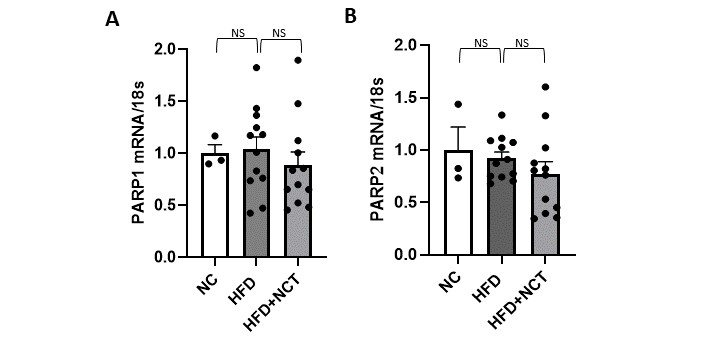


**Supplementary Figure 4: No difference in *Parp1* and *Parp2* mRNA expression: A-B.** qPCR analysis in mouse liver of *Parp1* and *Parp2* mRNA expression normalized with 18s (NC, N=3, HFD and NCT, N=12). Dots indicate individual mice. Values represent the mean ± SEM. NS=non-significant.

**Supplementary Table 1: Monitoring of NCT-treated mice**

| **Group**  **(n=6)** | **NCT Dose (mg/kg)** | **Duration of chow treatment** | **Post treatment monitoring** | **Symptoms observed** | | | | |
| --- | --- | --- | --- | --- | --- | --- | --- | --- |
|  |  |  |  |  | | | | |
| NCT | 200 | 10 weeks | 1 week | No | No | No | No | No |

**Supplementary Table 2: Hematological analysis**


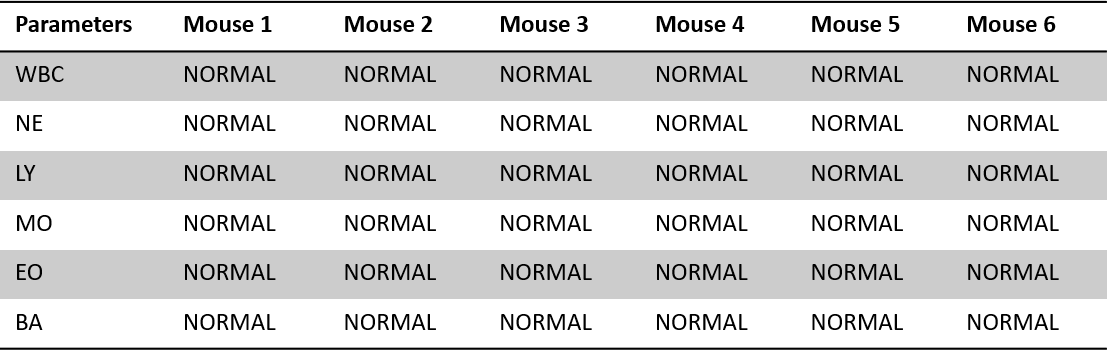

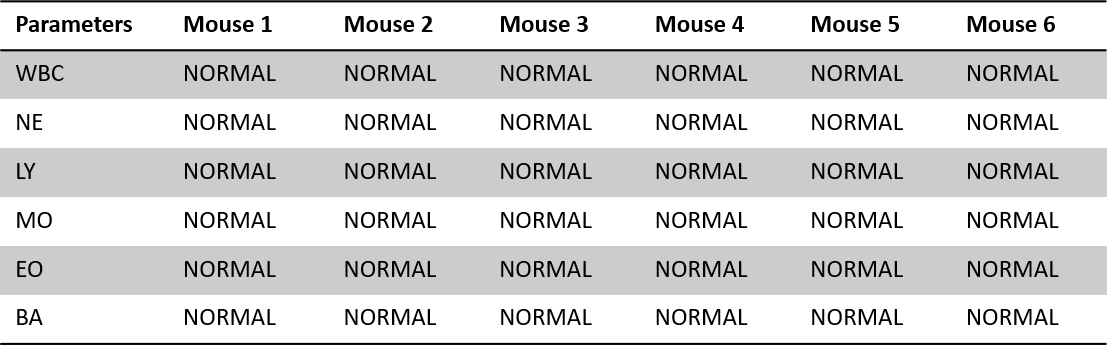


**HFD**

**NCT**

**Supplementary Table 3: Liver profile analysis**


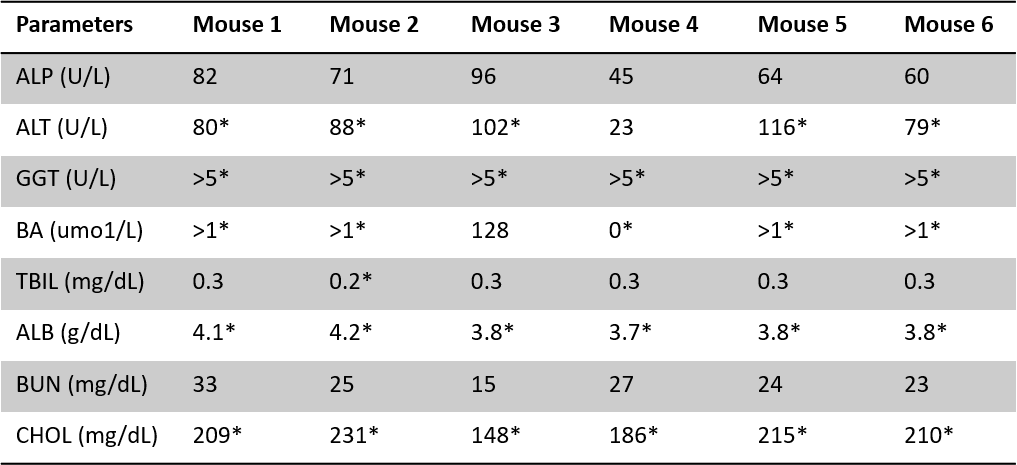

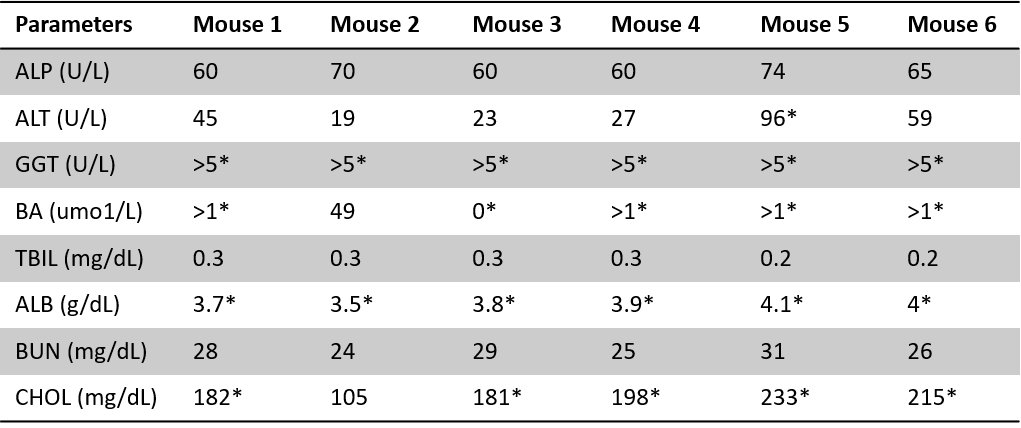


**HFD**

**NCT**
